# D-4F Ameliorates Contrast Media–Induced Oxidative Injuries in Endothelial Cells via the AMPK/PKC Pathway

**DOI:** 10.3389/fphar.2020.556074

**Published:** 2021-02-15

**Authors:** Yansong Guo, Wei Li, Mingming Qian, Ting Jiang, Ping Guo, Qian Du, Na Lin, Xianwei Xie, Zhiyong Wu, Donghai Lin, Donghui Liu

**Affiliations:** ^1^ Department of Cardiology, Fujian Provincial Hospital, Fujian Provincial Key Laboratory of Cardiovascular Disease, Fujian Cardiovascular Institute, Fujian Provincial Center for Geriatrics, Provincial Clinical Medicine College of Fujian Medical University, Fuzhou, China; ^2^ Department of Cardiology, the Affiliated Xiamen Cardiovascular Hospital of Xiamen University, Medical College of Xiamen University, Xiamen, China; ^3^ MOE Key Laboratory of Spectrochemical Analysis & Instrumentation, High-field NMR Research Center, College of Chemistry and Chemical Engineering, Xiamen University, Xiamen, China

**Keywords:** apolipoprotein A-I mimetic peptide, contrast media, endothelial cell, high-density lipoprotein, oxidative stress

## Abstract

Endothelial dysfunction is involved in the pathophysiological processes of contrast media (CM)–induced acute kidney injury (CI-AKI) after vascular angiography or intervention. Previous study found that apolipoprotein A-I (apoA-I) mimetic peptide, D-4F, alleviates endothelial impairments via upregulating heme oxygenase-1 (HO-1) expression and scavenging excessively generated reactive oxygen species (ROS). However, whether D-4F could ameliorate oxidative injuries in endothelial cells through suppressing ROS production remains unclear. In this study, a representative nonionic iodinated CM, iodixanol, was chosen for the *in vitro* and *in vivo* studies. Endothelial cell viability was assayed using micrographs, lactate dehydrogenase (LDH) activity, and cell counting kit-8 (CCK-8). Apoptosis was detected using flow cytometry analysis and caspase-3 activation. Endothelial inflammation was tested using monocyte adhesion assay and adhesion molecule expression. ROS production was detected by measuring the formation of lipid peroxidation malondialdehyde (MDA) through the thiobarbituric acid reactive substance (TBARS) assay. Peroxynitrite (ONOO⁻) formation was tested using the 3-nitrotyrosine ELISA kit. Iodixanol impaired cell viability, promoted vascular cell adhesion molecule-1 (VCAM-1) and intercellular cell adhesion molecule-1 (ICAM-1) expression, and induced cell apoptosis in human umbilical vein endothelial cells (HUVECs). However, D-4F mitigated these injuries. Furthermore, iodixanol induced the phosphorylation of protein kinase C (PKC) beta II, p47, Rac1, and endothelial nitric oxide synthase (eNOS) at Thr495, which elicited ROS release and ONOO⁻ generation. D-4F inhibited NADPH oxidase (NOX) activation, ROS production, and ONOO⁻ formation via the AMP-activated protein kinase (AMPK)/PKC pathway. Additionally, after an intravascular injection of iodixanol in Sprague Dawley rats, iodixanol induced a remarkable inflammatory response in arterial endothelial cells, although significant apoptosis and morphological changes were not observed. D-4F alleviated the vessel inflammation resulting from iodixanol *in vivo*. Collectively, besides scavenging ROS, D-4F could also suppress ROS production and ONOO⁻ formation through the AMPK/PKC pathway, which ameliorated oxidative injuries in endothelial cells. Hence, D-4F might serve as a potential agent in preventing CI-AKI.

## Introduction

Cardiovascular disease (CVD) causes disability and mortality worldwide (Joseph et al., 2017). Iodinated contrast media (CM) have become the most widely used reagents for diagnostic angiography and catheter-based intervention in patients with CVD (Fahling et al., 2017). The biocompatibility of CM is gradually improved; however, the growing use of CM still renders severe adverse effects (Seeliger et al., 2012). CM-induced acute kidney injury (CI-AKI) is the main iatrogenic complication after intravascular CM administration, which has been the third most common reason for new-onset renal failure in hospitalized patients (Seeliger et al., 2012; Fahling et al., 2017). The pathophysiological mechanisms underlying CI-AKI have not been fully elucidated, mainly including oxidative stress, inflammation, endothelial dysfunction, and renal tubular toxicity (Scoditti et al., 2013; Andreucci et al., 2014). Notably, CM are given through circulation during the diagnostic and interventional processes. These agents impair vascular endothelium and subsequently contribute to systemic and organ-specific adverse reactions (Scoditti et al., 2013). Therefore, endothelial cell impairments caused by CM have drawn attention as the key points in the pathogenesis of CI-AKI.

Endothelial cells play fundamental roles in maintaining vascular homeostasis. They control vascular tone, vessel inflammation, and thrombosis (Gimbrone and Garcia-Cardena, 2016; Bierhansl et al., 2017). After intravascular injection of CM, endothelial cells are exposed to CM in plasma, which induce endothelial dysfunction (Franke et al., 2008; Chang et al., 2014). CM increase the production of reactive oxygen species (ROS), the expression of vascular cell adhesion molecule-1 (VCAM-1), and the secretion of inflammatory factors (MCP-1, TNF-alpha, and IL-6) in endothelial cells (Ronda et al., 2013; Chang et al., 2014). Additionally, CM inhibit nitric oxide (NO) and prostacyclin (PGI2) release and promote endothelin-1 (ET-1) production in endothelial cells, which may be responsible for the vasoconstrictive response in renal microcirculation (Hutcheson et al., 1999; Zhao et al., 2011; Franke et al., 2012). Injection of CM in rats also changes the plasma levels of endothelium-related biomarkers, including plasminogen activator inhibitor-1 (PAI-1), tissue-type plasminogen activator (t-PA), and von Willebrand factor (vWF) (Ren et al., 2017). Furthermore, CM suppress endothelium-dependent arterial dilation in diabetic patients after angiography and trigger the release of circulating endothelial microparticles (EMPs) in both patients with CVD *in vivo* and endothelial cells *in vitro* (Xiang et al., 2014; Zhang et al., 2018). CM decrease cell viability and cause apoptosis and necrosis in endothelial cells, thus reducing blood flow velocity in renal capillaries and leading to renal medullary hypoxia and tubular failure (Franke et al., 2008; Zhao et al., 2009; Fahling et al., 2017). Consequently, these adverse effects on endothelial cells, such as apoptosis, inflammation, and vasoconstrictive responses, may be involved in the pathogenesis of CI-AKI (Scoditti et al., 2013). Therefore, understanding the pathogenic mechanisms underlying CI-AKI from endothelial impairments may help developing the novel preventive and therapeutic strategies for CI-AKI.

High-density lipoprotein (HDL) and its major apolipoprotein, apolipoprotein A-I (apoA-I), possess critical effects in correcting endothelial dysfunction (Feig et al., 2014; van Capelleveen et al., 2014). D-4F is one of the apoA-I mimetic peptides, which shares similar structure and function with native apoA-I and provides remarkable protection to endothelial cells (Navab et al., 2010). The metabolomic analysis demonstrated that D-4F alleviates oxidized low-density lipoprotein (ox-LDL)–induced oxidative stress and abnormal glycolysis in endothelial cells (Xu et al., 2019). D-4F promotes the activation of endothelial nitric oxide synthase (eNOS) and the generation of NO in coronary artery endothelial cells, thus protecting against ischemia/reperfusion injuries in mice (Baotic et al., 2013). Also, D-4F displays anti-oxidative, anti-inflammatory and antiapoptotic effects on endothelial cells through upregulating heme oxygenase-1 (HO-1) and scavenging excessively generated ROS (Liu et al., 2017a). However, whether D-4F could ameliorate CM-induced oxidative injuries in endothelial cells and which mechanisms were involved in these processes remained elusive.

This study showed that D-4F inhibited ROS production and peroxynitrite (ONOO⁻) formation, improved cell viability, and ameliorated apoptosis and inflammation induced by iodixanol in human umbilical vein endothelial cells (HUVECs) via the AMP-activated protein kinase (AMPK)/PKC pathway. Therefore, besides scavenging intracellular ROS, D-4F could also suppress ROS generation and ONOO⁻ formation and subsequently improve oxidative injuries resulting from CM in endothelial cells. Hence, D-4F might serve as a potential agent for preventing and treating CI-AKI.

## Materials and Methods

### Reagents and Antibodies

The study used primary antibodies against phospho-eNOS (Thr495, 9,574, Cell Signaling Technology, MA, USA), phospho-eNOS (Ser1177, 9,571, Cell Signaling Technology), eNOS (9,572, Cell Signaling Technology), phospho-Rac1 (2461, Cell Signaling Technology), VCAM-1 (12,367, Cell Signaling Technology), intracellular cell adhesion molecule 1 (ICAM-1) (4,915, Cell Signaling Technology), cleaved-caspase-3 (9,661, Cell Signaling Technology), phospho-PKC beta II (ab194749, Abcam, MA, USA), phospho-p47 (ab63554, Abcam), Rac1 (ab33186, Abcam), vWF (ab6994, Abcam), p47 (sc-365215, Santa Cruz Biotechnology, CA, USA), caspase-3 (BA2142, Boster, China), and PKC beta II (BM4261, Boster). Secondary antibodies were horseradish peroxidase (HRP)-goat anti-rabbit IgG (MBL458, MBL, Japan) and HRP-goat anti-mouse IgG (MBL 330, MBL). Compound C (S7306, Selleck Chemicals, TX, USA), VAS2870 (HY-12804, MedChemExpress, NJ, USA), L-NAME (HY-18729A, MedChemExpress), D-4F (purity 95%, China Peptide Company, China), iodixanol (Visipaque, 320 mg I/ml, GE Healthcare Company, IL, USA), Calcein-AM (KGMP012-1, Key GENBio TECH, Nanjing, China), lipid peroxidation (MDA) assay kit (MAK085, Sigma-Aldrich, MO, USA), Annexin V-FITC apoptosis detection kit (Dojindo Molecular Technologies, Japan), 3-nitrotyrosine ELISA kit (ab116691, Abcam, MA, USA), 3,3-diaminobenzidine (DAB) substrate kit (ab64238, Abcam), endothelial cell medium (ECM1001, ScienCell, CA, USA), and RPMI 1640 (Gibco Co., CA, USA) were used in the study. All other chemicals and reagents were of analytical grade and obtained from commercial sources.

### Cell Culture

HUVECs were obtained and cultured as mentioned before (Liu et al., 2011). HUVECs were used at passages 3–5. They were serum-deprived with 0.5% fetal bovine serum (FBS)-ECM for 6 h before further treatment. THP-1 monocytes (Cell Resource Center, Peking Union Medical College, China) were cultured as mentioned earlier (Liu et al., 2017a).

### Cell Viability Assay

HUVECs were pretreated with or without D-4F (20 μg/ml) for 8 h and then incubated with iodixanol (0, 5, 10, 30, or 60 mg I/ml) for 12 h. Cell photographs were taken, and cell numbers were counted in six random high-power fields (100×). HUVECs were preincubated with D-4F (0, 10, 20, or 50 μg/ml) for 8 h and subsequently treated with iodixanol (10 or 30 mg I/ml) for 12 h. Cell viability was evaluated using cell counting kit-8 (CCK-8) as mentioned earlier (Liu et al., 2017a). Lactate dehydrogenase (LDH) activity in the culture medium was tested using the LDH assay kit. All experiments were repeated three to four times.

### Apoptosis Assay

Apoptosis was assayed by flow cytometry analysis and caspase-3 activation. HUVECs were pretreated with or without D-4F (20 μg/ml) for 8 h and then incubated with iodixanol (0, 5, 10, 30, or 60 mg I/ml) for 12 h. Cell apoptosis was tested using an Annexin V-FITC apoptosis detection kit. Briefly, HUVECs were washed with PBS, resuspended in Annexin V binding buffer, and incubated with Annexin V-FITC and propidium iodide for 15 min at room temperature in the dark. Then, cells were analyzed by flow cytometry (Beckman Coulter, CA, USA) within 1 h. Alternatively, the activation of caspase-3 was detected through Western blot assay. All experiments were repeated three to four times.

### Monocyte–Endothelial Cell Adhesion Assay

The adhesion of THP-1 monocytes to endothelial cells was tested (Liu et al., 2017a). After reaching 80% confluence, endothelial cells were treated with or without D-4F (20 μg/ml) for 8 h and incubated with iodixanol (0, 5, 10, 30 or 60 mg I/ml) for 12 h. Subsequently, calcein AM–labeled THP-1 cells were overlaid on HUVECs and co-incubated for 1 h at 37°C. Non-adherent THP-1 cells were gently washed three times with PBS. Cell images (100×) were taken at 480 nm excitation and 530 nm emission using fluorescence microscopy (Leica, Germany). THP-1 monocytes attached to HUVECs were counted using Leica QWin analysis software. All experiments were repeated three to four times.

### Malondialdehyde (MDA) Assay

Intracellular ROS production was determined by detecting the formation of lipid peroxidation MDA through thiobarbituric acid reactive substance (TBARS) assay. HUVECs were preincubated with VAS2870 (2 μmol/l) for 30 min or L-NAME (1 mmol/L) for 1 h and then treated with iodixanol (30 mg I/ml) for 6 h. Alternatively, HUVECs were preincubated with or without Compound C (2 μmol/L) for 1 h. Then, HUVECs were treated in the absence or presence of D-4F (20 μg/ml) for 8 h and further incubated with iodixanol (30 mg I/ml) for 6 h. Subsequently, cells were washed twice with PBS and lysed with lysis buffer. After centrifugation, the supernatant was used for MDA assay following the manufacturer’s instructions. The absorbance was measured at 532 nm using a microplate reader (SpectraMax® iD5, Molecular Devices, CA, USA). Protein concentrations were determined by BCA protein assay kit. All experiments were repeated three times.

### Peroxynitrite (ONOO⁻) Assay

Peroxynitrite (ONOO⁻) formation was assayed through determining the production of 3-nitrotyrosine using the ELISA kit. HUVECs were treated as described above. After cell lysis, the centrifuging supernatant was used for 3-nitrotyrosine assay according to the manufacturer’s protocol. A microplate reader was used to measure the absorbance at 450 nm. Protein concentrations were measured by BCA protein assay kit. All experiments were repeated three times.

### Western Blot Analysis

The activation of caspase-3 and the expression of VCAM-1 and ICAM-1 were tested by pretreating HUVECs with or without D-4F (20 μg/ml) for 8 h and incubating with iodixanol (0, 5, 10, 30, or 60 mg I/ml) for 12 h. HUVECs were treated with iodixanol (30 mg I/ml) for 0, 5, 15, 30, 60, and 120 min to investigate the phosphorylation of PKC beta II, p47, Rac1, eNOS (Thr495), as well as eNOS (Ser1177). Whether AMPK was involved in the protection of D-4F was found out by preincubating HUVECs with or without Compound C (2 μmol/L) for 1 h. Subsequently, HUVECs were incubated in the absence or presence of D-4F (20 μg/ml) for 8 h and further stimulated with iodixanol (30 mg I/ml) for 30 min to detect the activation of PKC beta II, p47, Rac1, and eNOS (Thr495). All experiments were repeated three to four times.

### Animals and Grouping

Male Sprague Dawley rats (300-350 g) were purchased from the Experimental Animal Center of Xiamen University and acclimatized for 7 days before studies according to the Guide for the Care and Use of Laboratory Animals published by the US National Institutes of Health (Publication No. 85-23, revised 1996). These rats were randomly divided into three groups: 1) control (*n* = 10), 2) iodixanol (*n* = 10), and 3) D-4F + iodixanol (*n* = 10). D-4F (1 mg/kg body weight) or equal volumes of PBS was given intragastricallystarting 2 days prior to iodixanol injection and continued daily. All rats were injected with iodixanol (4 g I/kg body weight) or equal volumes of PBS via the tail vein once after D-4F treatment (Liu et al., 2017b; Ren et al., 2017). The rats were euthanized by an overdose of sodium pentobarbital (150 mg/kg body weight) administered by intraperitoneal injection and sacrificed after 24 h. The carotid arteries were harvested and processed for morphological studies.

### Histomorphometry and Immunohistochemistry

The carotid arteries were fixed with 4% paraformaldehyde and paraffin-embedded. Serial 5-μm sections were cut (Leica Microsystems, Germany) and stained with hematoxylin and eosin (H&E). The integrity and inflammatory responses in arterial endothelial cells were determined by testing the expression of vWF and ICAM-1 through immunohistochemistry. Briefly, the sections were deparaffinized, rehydrated, and heated in 0.01 mol/L sodium citrate buffer (pH 6.0) for 10 min. Then, the sections were blocked with 5% goat serum in PBS for 1 h before they were incubated with primary antibodies to vWF (1:100) and ICAM-1 (1:100) overnight at 4°C. Subsequently, the sections were exposed to biotinylated secondary goat anti-rabbit antibody (1:1,000) for 1 h and to streptavidin-HRP (1:500) for 15 min at room temperature. Following this procedure, the sections were washed three times with PBS and developed using the DAB substrate kit. The nuclei were counterstained with hematoxylin. And the sections were photographed (1,000×) using microscopy (Leica, Germany).

### Statistical Analysis

Differences were compared with the two-tailed Student *t* test or one-way analysis of variance using GraphPad Prism (version 6.0, La Jolla, CA, USA). Data were expressed as mean ± standard error of mean. A *p* value less than 0.05 (*p* < 0.05) was considered statistically significant (*p < 0.05, **p < 0.01, *p* < 0.001).

## Results

### D-4F Improved the Viability of Human Umbilical Vein Endothelial Cells Impaired by Iodixanol

**Figure 1:**
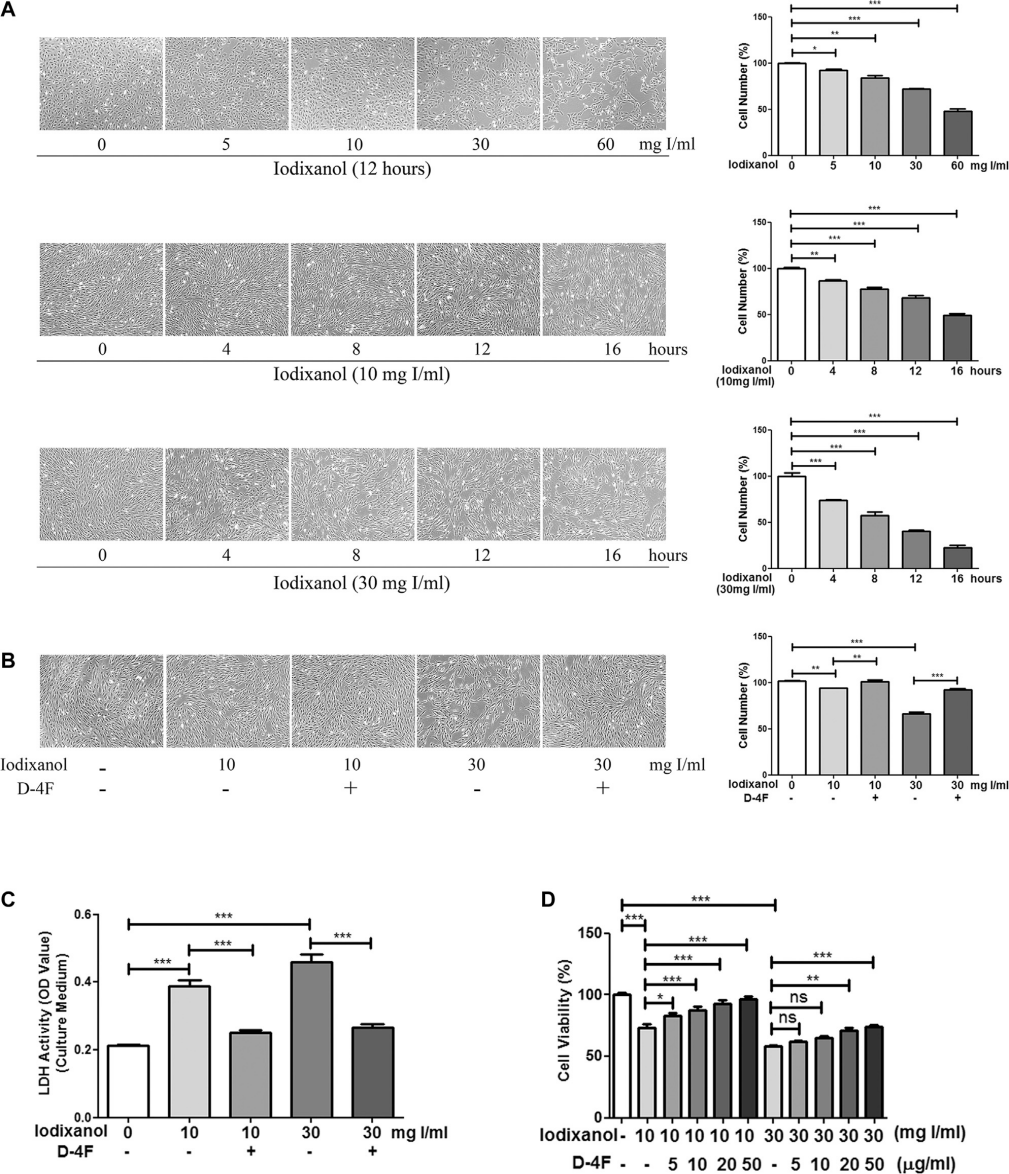
D-4F improved the viability of HUVECs impaired by iodixanol. HUVECs were treated with iodixanol (0, 5, 10, 30, or 60 mg I/ml) for 12 h or with iodixanol (10 or 30 mg I/ml) for 0, 4, 8, 12, or 16 h **(A)**. Alternatively, HUVECs were preincubated with or without D-4F (20 μg/ml) for 8 h and further treated with iodixanol (10 or 30 mg I/ml) for 12 h **(B,C)**. The cells were photographed and counted in six random high-power fields (100×) **(A,B)**. LDH activity in the culture medium was tested using a LDH assay kit **(C)**. HUVECs were preincubated with D-4F (0, 5, 10, 20, or 50 μg/ml) for 8 h and then treated with iodixanol (10 or 30 mg I/ml) for 12 h. Cell viability was tested using a CCK-8 kit **(D)**.

Cell vitality was tested through morphological studies, CCK-8 measurement, and LDH activity assay. Iodixanol decreased the viability of HUVECs in the dose- and time-dependent manners (Figure 1A), and D-4F improved these injuries caused by iodixanol (Figure 1B). Meanwhile, D-4F reduced the elevated LDH activity resulting from iodixanol in the culture medium (Figure 1C), indicating a decrease in cell impairments. Besides, CCK-8 assay also yielded the similar results, indicating that D-4F alleviated the iodixanol-impaired vitality of HUVECs in a dose-dependent manner (Figure 1D).

### D-4F Alleviated Iodixanol-Induced Apoptosis of Human Umbilical Vein Endothelial Cells

**Figure 2:**
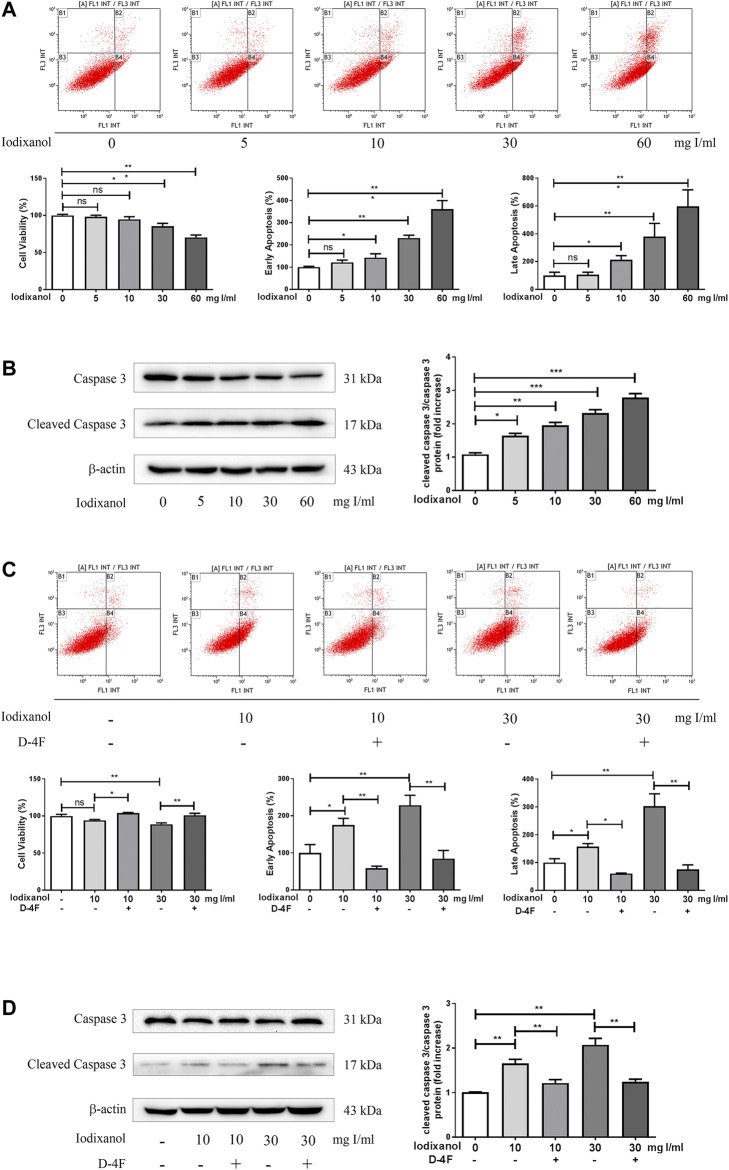
D-4F alleviated iodixanol-induced apoptosis of HUVECs. HUVECs were treated with iodixanol (0, 5, 10, 30, or 60 mg I/ml) for 12 h **(A,B)**. Alternatively, HUVECs were preincubated with or without D-4F (20 μg/ml) for 8 h and further treated with iodixanol (10 or 30 mg I/ml) for 12 h **(C,D)**. Cell apoptosis was detected by flow cytometry analysis **(A,C)**, and caspase-3 activation was assayed by Western blot analysis **(B,D)**.

Cell apoptosis was assayed through flow cytometry analysis and caspase-3 activation. Iodixanol reduced the viability of HUVECs and increased early apoptosis and late apoptosis in a dose-dependent manner (Figure 2A). Meanwhile, iodixanol promoted caspase-3 activation in a dose-dependent manner (Figure 2B). Nevertheless, D-4F decreased iodixanol-triggered apoptosis of HUVECs, as shown by flow cytometry assay (Figure 2C) and Western blot analysis (Figure 2D).

### D-4F Suppressed the Inflammatory Responses Caused by Iodixanol in Human Umbilical Vein Endothelial Cells

**Figure 3:**
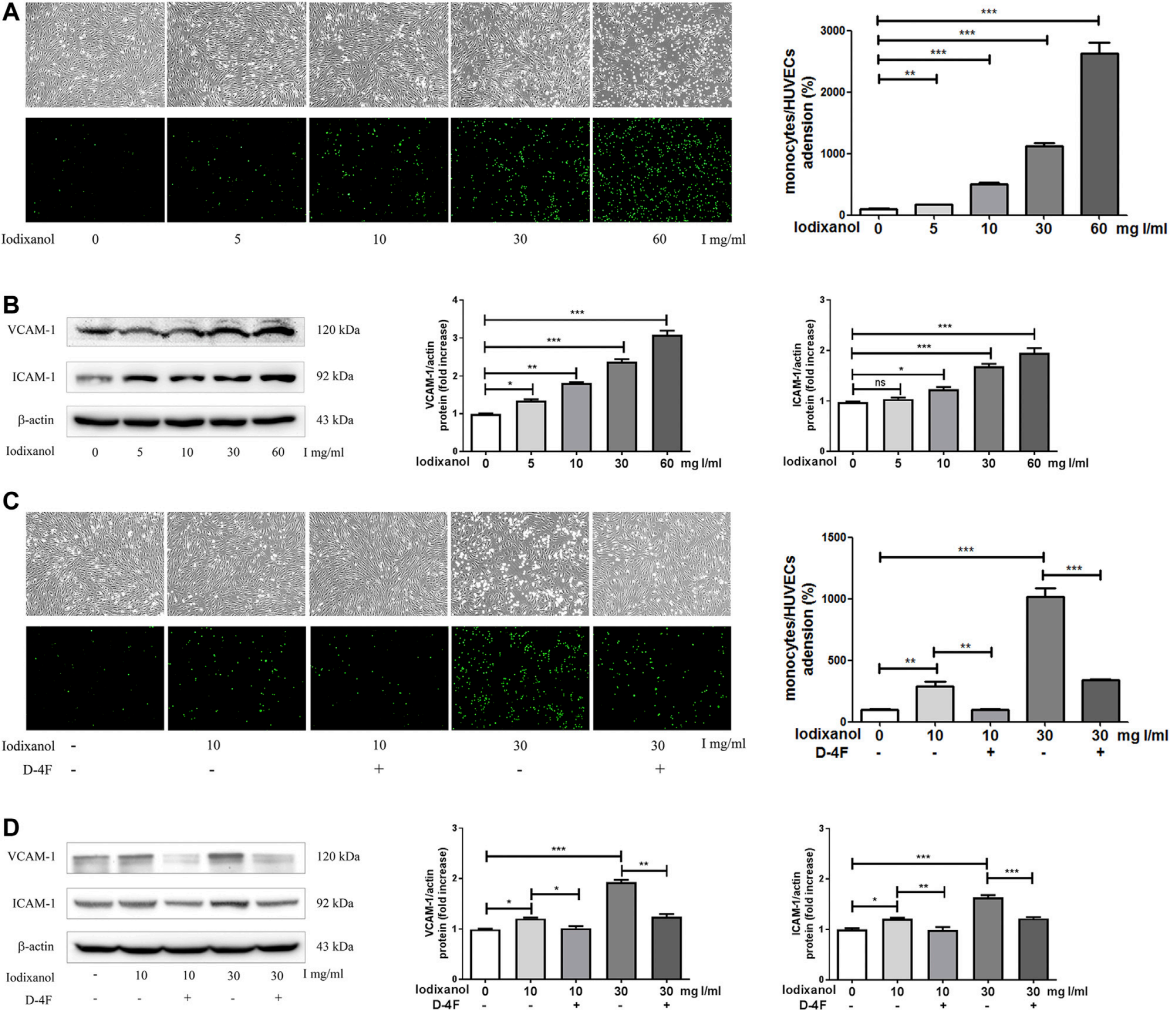
D-4F suppressed the inflammatory responses caused by iodixanol in HUVECs. HUVECs were treated with iodixanol (0, 5, 10, 30, or 60 mg I/ml) for 12 h **(A,B)**. Alternatively, HUVECs were preincubated with or without D-4F (20 μg/ml) for 8 h and then treated with iodixanol (10 or 30 mg I/ml) for 12 h **(C,D)**. HUVECs were washed with PBS and incubated with fluorescence-labeled THP-1 monocytes (1 × 10^6^ cell/well) for 30 min. Fluorescence numbers were counted (100×) **(A,C)**. The expression of VCAM-1 and ICAM-1 were assayed by Western blot analysis **(B,D)**.

The adhesion of monocytes to endothelial cells was tested by fluorescence microscopy, and the expression of VCAM-1 and ICAM-1 were assayed using Western blot analysis. Iodixanol significantly increased the adhesion of fluorescence-labeled THP-1 monocytes to HUVECs and upregulated the expression of VCAM-1 and ICAM-1 in a dose-dependent manner (Figures 3A, B). However, D-4F mitigated the increased adhesion of THP-1 monocytes to HUVECs and the upregulation of VCAM-1 and ICAM-1 induced by iodixanol (Figures 3C, D).

### Iodixanol Triggered Protein Kinase C Phosphorylation, NADPH Oxidase Activation, and Endothelial Nitric Oxide Synthase Dysregulation in Human Umbilical Vein Endothelial Cells

**Figure 4:**
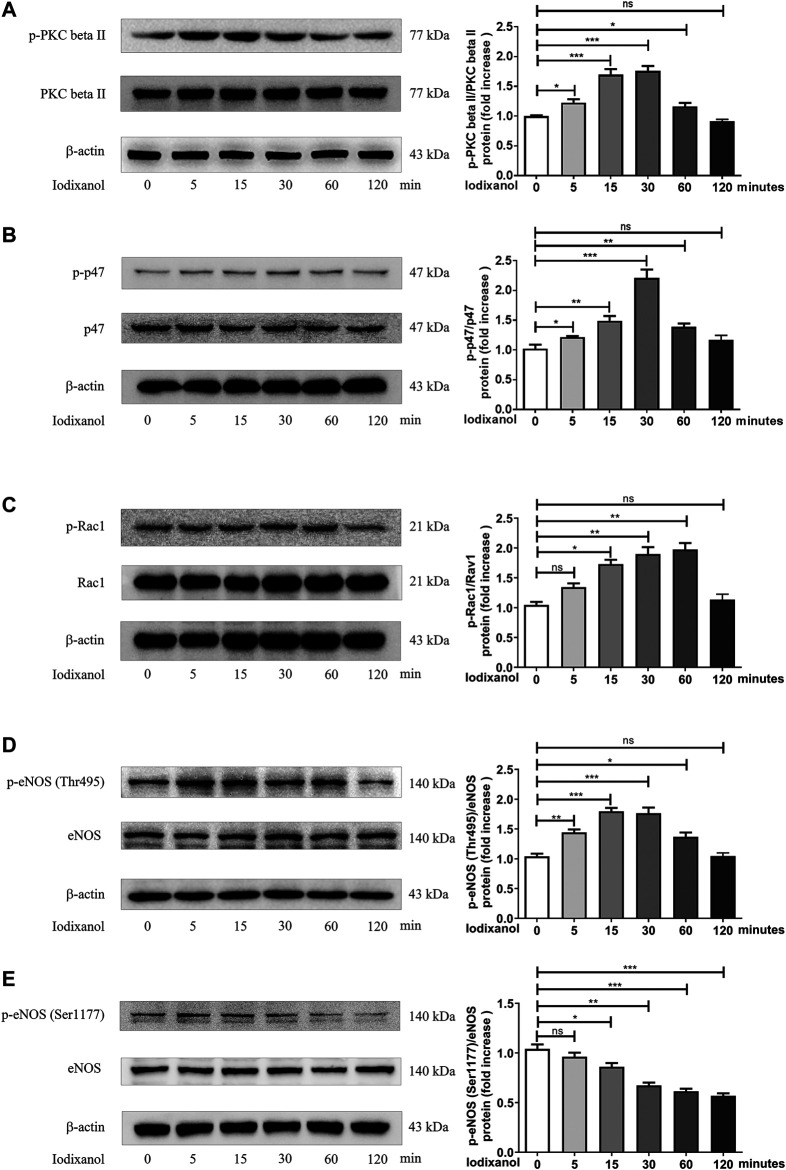
Iodixanol triggered PKC phosphorylation, NOX activation, and eNOS dysregulation in HUVECs. HUVECs were incubated with iodixanol (30 mg I/ml) for 0, 5, 15, 30, 60, or 120 min. The phosphorylation of PKC beta II, p47, Rac1, eNOS (Thr495), and eNOS (Ser1177) was assayed by Western blot analysis **(A-E)**.

PKC is involved in the activation of NOX and the dysregulation of eNOS (Deng et al., 2012; Yan et al., 2017). The phosphorylation of PKC beta II, p47, Rac1, eNOS (Thr495), and eNOS (Ser1177) was tested to determine whether NOX activation and eNOS dysregulation took part in oxidative stress caused by CM in endothelial cells. The results showed that iodixanol increased the phosphorylation of PKC beta II, p47, Rac1, and eNOS (Thr495) and decreased the phosphorylation of eNOS (Ser1177) in HUVECs (Figures 4A–E). Thus, it appeared that iodixanol triggered oxidative injuries in endothelial cells through NOX activation and eNOS dysregulation via the PKC pathway. Additionally, iodixanol also upregulated the expression of NOX2 and NOX4 and reduced the generation of NO in a time-dependent manner in HUVECs (Supplementary Figures 1, 2).

### NADPH Oxidase Activation and Endothelial Nitric Oxide Synthase Dysregulation Were Involved in Iodixanol-Induced Oxidative Stress in Human Umbilical Vein Endothelial Cells

**Figure 5:**
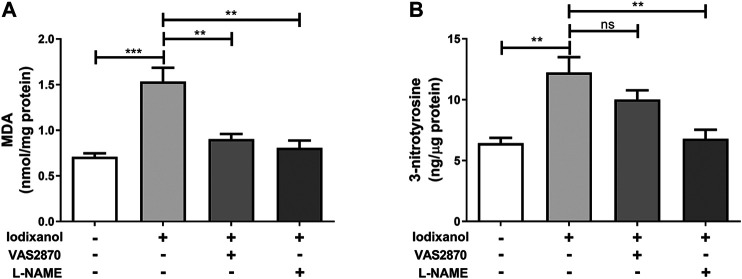
NOX activation and eNOS dysregulation were involved in iodixanol-induced oxidative stress in HUVECs. HUVECs were preincubated with VAS2870 (2 μmol/L) for 30 min or L-NAME (1 mmol/L) for 1 h and further treated with iodixanol (30 mg I/ml) for 6 h. MDA formation was determined using a TBARS assay kit **(A)**. 3-nitrotyrosine production was tested using an ELISA kit **(B)**.

Intracellular ROS generation was detected by monitoring the formation of lipid peroxidation MDA. ONOO⁻ formation was assayed through testing the production of 3-nitrotyrosine. Iodixanol significantly increased MDA formation and 3-nitrotyrosine production in HUVECs. Inhibition of NOX (VAS2870) and eNOS (L-NAME) repressed MDA formation triggered by iodixanol (Figure 5A). Besides, L-NAME inhibited iodixanol-induced 3-nitrotyrosine production (Figure 5B). Although VAS2870 seemed to decrease 3-nitrotyrosine levels, it did not obtain the statistically significant.

### D-4F Inhibited Iodixanol-Induced NADPH Oxidase Activation and Endothelial Nitric Oxide Synthase Dysregulation through the AMP-Activated Protein Kinase/Protein Kinase C Pathway in Human Umbilical Vein Endothelial Cells

**Figure 6:**
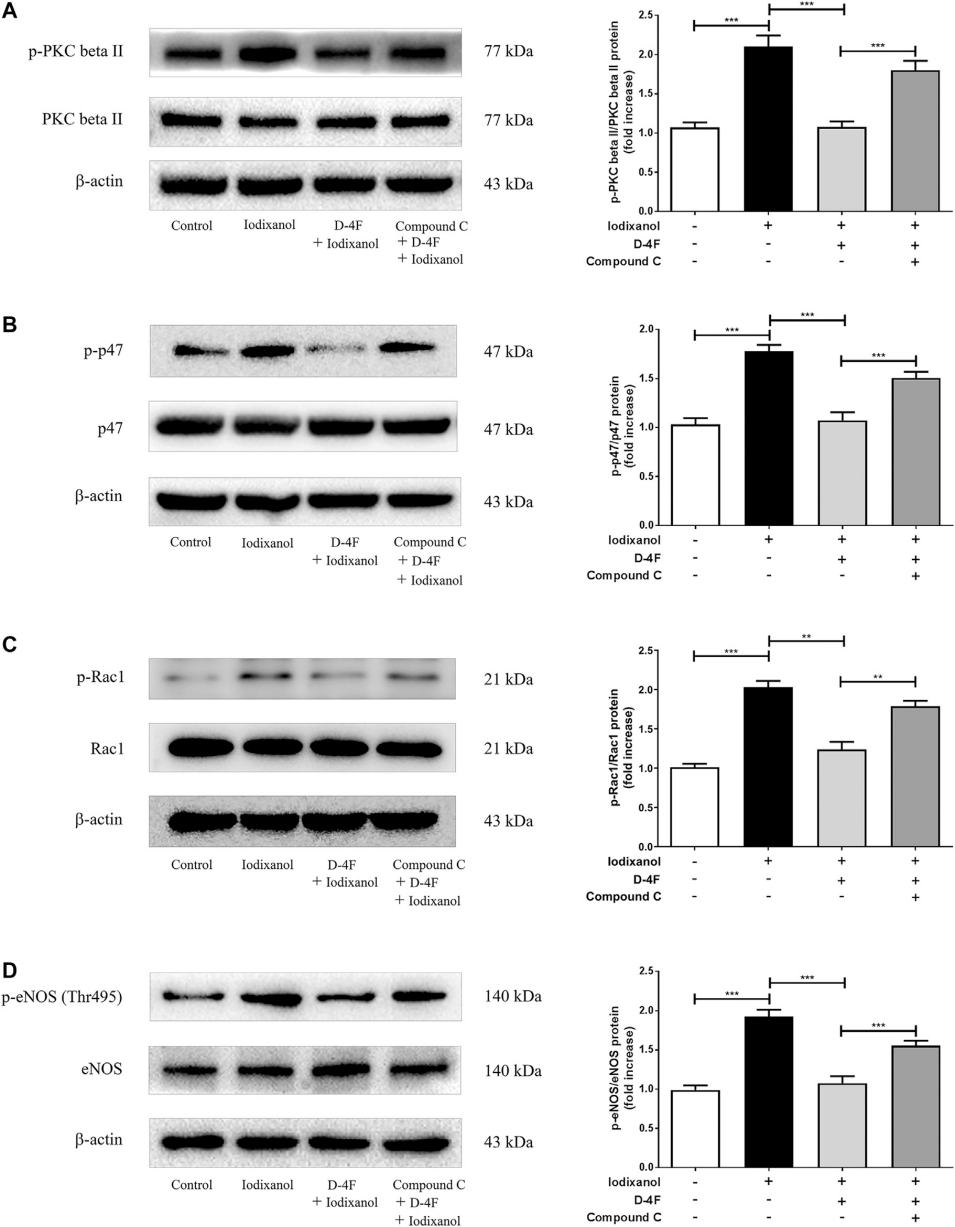
D-4F inhibited iodixanol-induced NOX activation and eNOS dysregulation through the AMPK/PKC pathway in HUVECs. HUVECs were preincubated in the absence or presence of Compound C (2 μmol/L) for 1 h. Then, cells were incubated with or without D-4F (20 μg/ml) for 8 h and treated with iodixanol (30 mg I/ml) for 30 min. The phosphorylation of PKC beta II, p47, Rac1, and eNOS (Thr495) was assayed by Western blot analysis **(A-D)**.

Previous study demonstrated that D-4F upregulates HO-1 expression via the AMPK-dependent pathway (Liu et al., 2017a). An AMPK inhibitor, Compound C, was employed. The phosphorylation of PKC beta II, p47, Rac1, and eNOS (Thr495) was analyzed by Western blot to investigate whether AMPK was also involved in the protections of D-4F against CM-induced ROS production in endothelial cells. D-4F inhibited the phosphorylation of PKC beta II, p47, Rac1, and eNOS (Thr495); however, Compound C eliminated these inhibitory effects of D-4F on the activation of iodixanol-triggered PKC beta II, p47, Rac1, and eNOS (Thr495) in HUVECs (Figures 6A–D). Thus, it appeared that D-4F decreased iodixanol-triggered NOX activation and eNOS dysregulation through the AMPK/PKC pathway.

### D-4F Repressed Iodixanol-Triggered Oxidative Stress Through the AMP-Activated Protein Kinase–Dependent Pathway in Human Umbilical Vein Endothelial Cells

**Figure 7:**
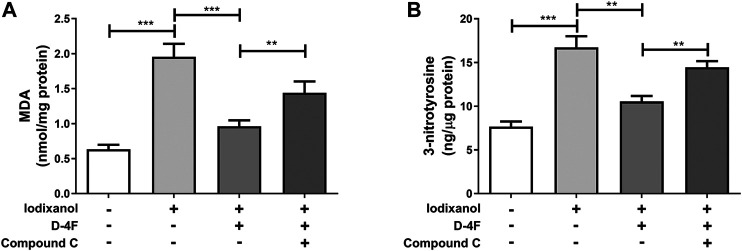
D-4F repressed iodixanol-triggered oxidative stress through the AMPK-dependent pathway in HUVECs. HUVECs were preincubated with or without Compound C (2 μmol/L) for 1 h and subsequently treated with D-4F (20 μg/ml) for 8 h. Then, HUVECs were further incubated with iodixanol (30 mg I/ml) for 6 h. MDA formation was determined using a TBARS assay kit **(A)**. 3-nitrotyrosine production was tested using an ELISA kit **(B)**.

Production of ROS was determined through MDA assay. Formation of ONOO⁻ was tested by 3-nitrotyrosine measurement. Iodixanol induced MDA formation and 3-nitrotyrosine production in HUVECs, and D-4F antagonized the increase of MDA and 3-nitrotyrosine triggered by iodixanol. However, inhibition of AMPK by Compound C decreased the inhibitory effects of D-4F on MDA formation and 3-nitrotyrosine production (Figures 7A, B). Additionally, we also directly detected ROS release by monitoring the fluorescence intensity from 2′,7′-dichlorofluorescin diacetate (DCFH-DA) oxidation. The similar results showed that D-4F repressed iodixanol-triggered ROS production in HUVECs, and Compound C eliminated the anti-oxidative effects of D-4F (Supplementary Figure 3).

### D-4F Ameliorated Iodixanol-Induced Inflammatory Responses in Vascular Endothelial Cells in Rats

**Figure 8:**
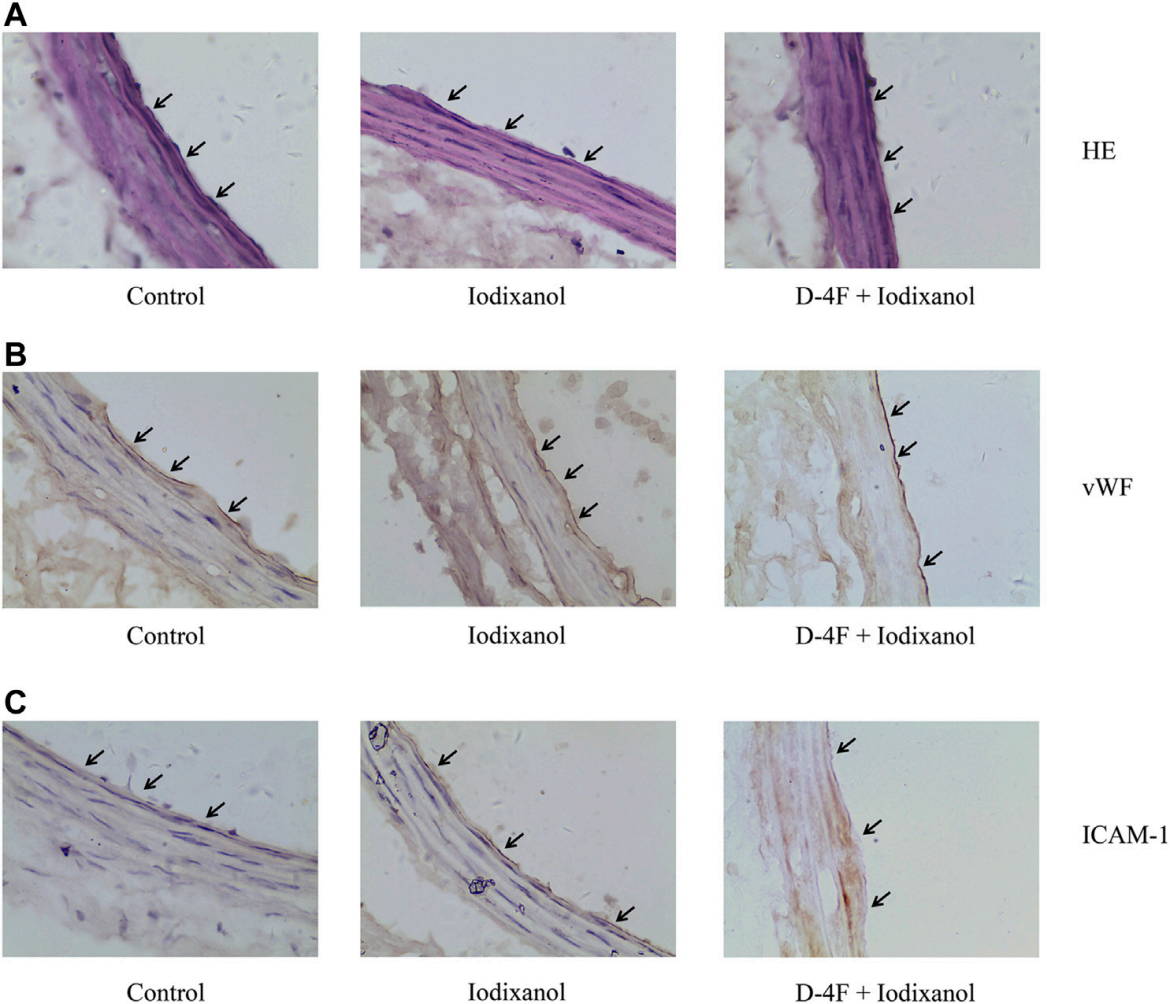
D-4F ameliorated iodixanol-induced inflammatory responses in vascular endothelial cells in rats. Representative micrographs with H&E staining **(A)** and immunohistochemical staining for vWF **(B)** and ICAM-1 **(C)** of carotid artery sections in Sprague Dawley rats were shown (1,000×).

Iodixanol was injected into Sprague Dawley rats to detect whether D-4F improved the inflammatory responses in endothelial cells *in vivo*. The vessels of carotid arteries were tested by H&E staining (Figure 8A) and immunohistochemistry for vWF and ICAM-1 (Figures 8B, C). Black arrows aimed at endothelial cells of vessel wall in carotid arteries. Detachment of endothelial cells was not observed in the vessel wall (Figure 8A). The expression of vWF was positive in carotid arteries in all three groups, indicating that the vessel endothelium was intact in these groups (Figure 8B). However, the expression of ICAM-1 was positive in endothelial cells in the iodixanol group and negative in the control and D-4F + iodixanol groups (Figure 8C), implying that D-4F inhibited the inflammatory responses resulting from iodixanol. Therefore, D-4F ameliorated CM-induced inflammation in vessel endothelium *in vivo*. In addition, we also found that D-4F decreased MDA formation in the kidneys (Supplementary Figure 4) and improved the morphological impairments in renal tubules triggered by iodixanol (Supplementary Figure 5), implying the protective effects of D-4F in the kidneys *in vivo*.

### Schematic of Molecular Mechanisms of D-4F against CM-Induced Oxidative Injuries in Endothelial Cells

**Figure 9:**
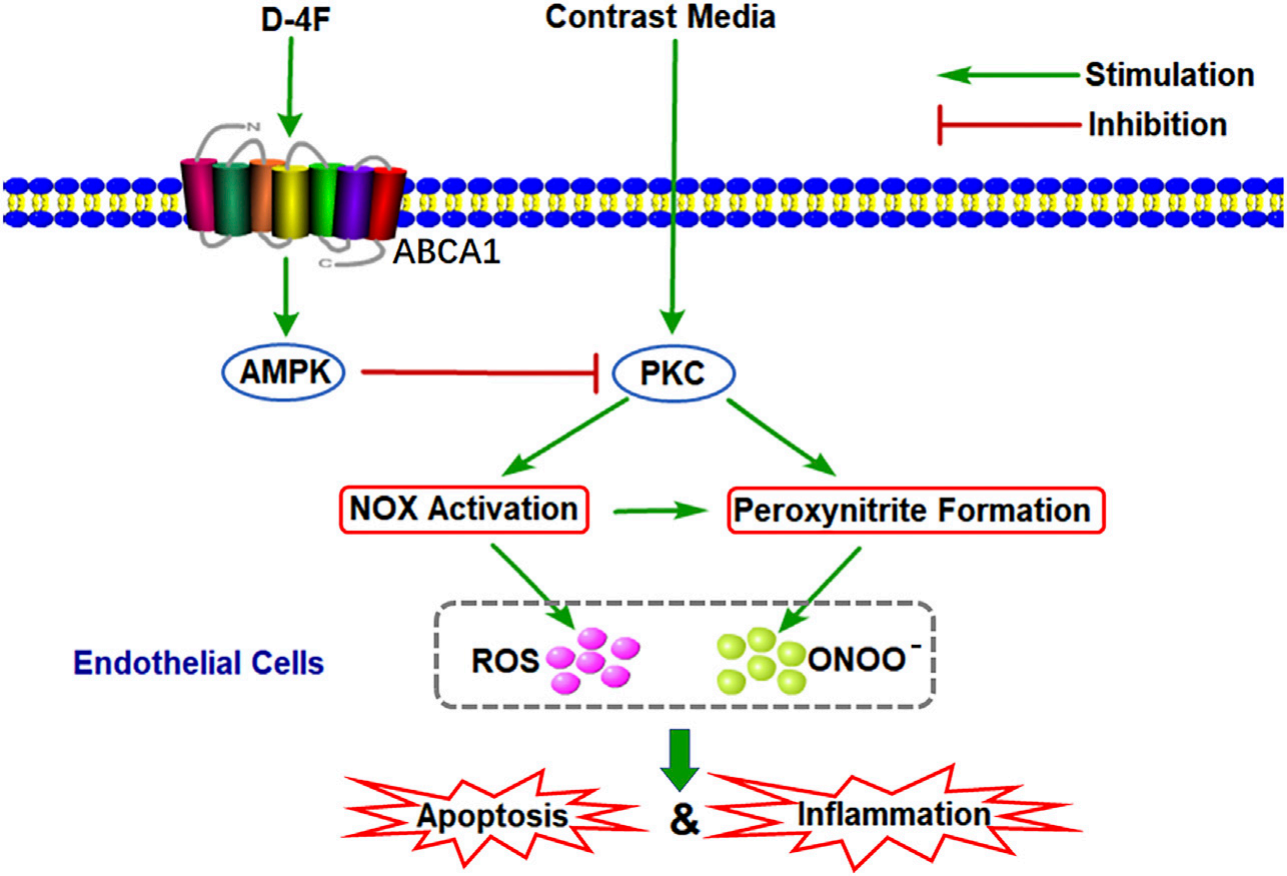
Schematic of molecular mechanisms of D-4F against CM-induced oxidative injuries in endothelial cells. D-4F repressed PKC phosphorylation and subsequently inhibited NOX activation and eNOS dysregulation through the AMPK-dependent pathway, thus reducing ROS production and ONOO⁻ formation and protecting endothelial cells from apoptosis and inflammation induced by CM.

The possible mechanisms by which D-4F inhibited CM-caused oxidative injuries in endothelial cells were shown in Figure 9. D-4F repressed PKC phosphorylation and subsequently inhibited NOX activation and eNOS dysregulation through the AMPK-dependent pathway, thus reducing ROS production and ONOO⁻ formation and protecting endothelial cells from apoptosis and inflammation induced by CM.

## Discussion

Iodinated CM could trigger oxidative stress and induce endothelial impairments (Scoditti et al., 2013; Chang et al., 2014). Our previous study demonstrated that D-4F improves endothelial dysfunction through upregulating HO-1 and scavenging excessively generated ROS (Liu et al., 2017a). However, whether D-4F could alleviate oxidative injuries through inhibiting ROS production in endothelial cells is still unclear. This study found that iodixanol induced NOX activation and eNOS dysregulation and promoted ROS generation and ONOO⁻ formation, eliciting proapoptotic and pro-inflammatory effects in endothelial cells. And D-4F inhibited ROS production and ONOO⁻ formation via the AMPK/PKC pathway, decreased iodixanol-induced apoptosis and inflammation.

Vasculopathy with functionally impaired endothelial cells is a hallmark of CI-AKI (Scoditti et al., 2013). CM inhibit vasodilator (NO and PGI2) production and promote vasoconstrictor (ET-1) generation in endothelial cells, consequently leading to renal vasoconstriction and tissue ischemia in the kidneys (Hutcheson et al., 1999; Zhao et al., 2011; Franke et al., 2012; Sendeski et al., 2012; Fahling et al., 2017). In addition, endothelial dysfunction decreases the anti-inflammatory and antithrombotic properties of vessels and promotes the development of systemic and organ-specific complications owing to CM administration (Chang et al., 2014). After CM injection in rats, the plasma levels of endothelial cell markers (vWF and t-PA) significantly increase (Ren et al., 2017). Furthermore, CM also induce the release of circulating EMPs in patients with CVD after angiography (Zhang et al., 2018). Therefore, improving endothelial dysfunction might be a useful strategy for preventing CI-AKI.

Oxidative stress is considered to be vital in the pathogenesis of endothelial dysfunction and CI-AKI (Scoditti et al., 2013; Andreucci et al., 2014). Once formed, CM-mediated ROS may induce apoptosis and inflammation in endothelial cells, thereby intensifying renal hypoxia and oxidative injuries (Ronda et al., 2013; Zhang et al., 2018). This study also found that iodixanol reduced cell viability and induced apoptosis and inflammation in endothelial cells (Figures 1–3). Overexpression of recombinant manganese superoxide dismutase (rMnSOD) could decrease oxidative stress in the kidneys and prevent the reduction of glomerular filtration rate after CM administration in rats (Pisani et al., 2014). Additionally, upregulation of thioredoxin-1 (Trx1) could diminish apoptosis and reduce the histological changes in renal tubules in CM-mediated kidney injuries (Gong et al., 2016). HO-1 could counteract the adverse effects of CM through ameliorating oxidative stress (Chang et al., 2014). Epigallocatechin-3-gallate improves CI-AKI by upregulating HO-1 and alleviating oxidative impairments and inflammation in rats (Gao et al., 2016). Previously, we demonstrated that D-4F upregulates HO-1 expression, decreases oxidative stress, and improves endothelial dysfunction (Liu et al., 2017a). The present study indicated that D-4F remarkably ameliorated iodixanol-induced apoptosis and inflammation in endothelial cells (Figures 1–3). Besides, we also found that iodixanol notably induced endothelial inflammation in rats *in vivo*, and orally administered D-4F improved the inflammatory responses of artery vessels caused by iodixanol (Figure 8). Some studies reported that injecting CM in rats does not induce apoptosis and necrosis in endothelial cells *in vivo* (Zhao et al., 2011). In this study, no obvious morphological changes and apoptosis were found in arterial endothelial cells *in vivo*, and the vascular endothelium was still intact with vWF staining after iodixanol injection in rats (Figure 8). Furthermore, D-4F also decreased MDA formation in the kidneys and improved the morphological impairments in renal tubules triggered by iodixanol (Supplementary Figures 4, 5). Thus, it was supposed that D-4F, as a powerful protector of endothelial cells, might be a potential agent against CI-AKI *in vivo*.

NOX activation is the main source of ROS in endothelial cells (Luo et al., 2014; Meza et al., 2019). Hyperglycemia increases NOX activity, ROS release, and cell apoptosis, and NOX inhibition abolishes the pro-oxidative and proapoptotic effects of hyperglycemia in endothelial cells (Shao and Bayraktutan, 2014). *Tert*-butyl hydroperoxide induces ROS generation, LDH release, and caspase-dependent apoptosis through NOX activation, which could be repressed through NOX inhibition (Zhao et al., 2017). Some studies reported that iodixanol upregulates NOX4 expression and increases ROS generation in renal tubular cells, inducing tubular cell apoptosis and necrosis (Netti et al., 2014). Also, iodixanol induced NOX activation, upregulated NOX2 and NOX4 expression, and triggered MDA formation; however, inhibition of NOX reduced oxidative stress in endothelial cells (Figures 4, 5, and Supplementary Figure 1).

eNOS is a homodimer that binds many different cofactors, converting L-arginine and O_2_ to L-citrulline and NO (Forstermann and Sessa, 2012). Aberrant NOX activation could promote eNOS dysregulation and uncoupling, leading to NO bioavailability reduction, superoxide generation, and ONOO⁻ formation, which create a toxic cycle of oxidative stress and consequently aggravate endothelial dysfunction (Lee et al., 2017; Meza et al., 2019; Siragusa and Fleming, 2016). Nicotine induces arterial stiffness in mice through ONOO⁻-mediated sirtuin-1 (SIRT1) inactivation (Ding et al., 2019). 1-Palmitoyl-2-(5-oxovaleroyl)-sn-glycero-3-phosphocholine (POVPC) induces eNOS uncoupling, including unbalanced Ser1177/Thr495 phosphorylation, decreased NO release, and elevated ROS generation, which consequently cause apoptosis in endothelial cells (Yan et al., 2017). CM could repress NO production in isolated arteries *in vitro* and reduce NO levels in plasma following angiography *in vivo* (Hutcheson et al., 1999; Murakami et al., 2002; Zhao et al., 2011). This study found that iodixanol caused abnormal eNOS (Ser1177/Thr495) phosphorylation, decreased NO generation, and increased ONOO⁻ formation in endothelial cells, and inhibition of eNOS could reduce MDA formation and 3-nitrotyrosine production (Figures 4, 5 and Supplementary Figure 2). Hence, CM-induced eNOS dysregulation, switching from an anti-oxidative phenotype to a pro-oxidative phenotype, seemed to play important roles in endothelial dysfunction.

PKC controls NOX activation and eNOS uncoupling (Deng et al., 2012; Yan et al., 2017). Both TNF-alpha and hyperglycemia promote ROS release and cell apoptosis through PKC-dependent NOX activation. However, inhibition or downregulation of PKC improves TNF-alpha- and hyperglycemia-caused oxidative stress and apoptosis in endothelial cells (Deng et al., 2012; Shao and Bayraktutan, 2014). POVPC induces oxidative impairments in endothelial cells through PKC-dependent eNOS uncoupling (Yan et al., 2017). ET-1 triggers eNOS phosphorylation at Thr495 via the PKC pathway (Sun et al., 2014). We also found that iodixanol induced PKC phosphorylation, which was consistent with NOX activation and eNOS phosphorylation (Thr495) caused by iodixanol in endothelial cells (Figure 4).

AMPK has a pivotal effect in regulating redox balance in endothelial cells (Shirwany and Zou, 2014). HDL attenuates ox-LDL-induced endothelial dysfunction through the AMPK-dependent pathway (Fisslthaler and Fleming, 2009; Valente et al., 2014). We found that D-4F inhibits oxidative stress and improves inflammation and apoptosis in endothelial cells via AMPK-dependent HO-1 upregulation (Liu et al., 2017a). AMPK is a physiological suppressor of NOX in endothelial cells (Song and Zou, 2012). Hyperhomocysteinemia (Hhcy) increases NOX activation through repressing SIRT1 and AMPK, inducing endothelial inflammation and apoptosis. However, exercise training protects endothelial cells against oxidative injuries through upregulating SIRT1 and AMPK (Chan et al., 2018). Metformin, an AMPK activator, could ameliorate high glucose-induced oxidative stress in endothelial cells through inhibiting the PKC-beta II/NOX pathway (Batchuluun et al., 2014; Gallo et al., 2005). Aspirin attenuates vinorelbine-induced PKC phosphorylation, NOX activation, and ROS production, subsequently preventing endothelial dysfunction via the SIRT1/AMPK axis (Tsai et al., 2014). The *Ginkgo biloba* extract protects endothelial cells against ox-LDL-induced PKC phosphorylation and NOX activation via the AMPK-dependent mechanisms (Ou et al., 2013). This study found that D-4F repressed iodixanol-induced PKC phosphorylation, NOX activation, and eNOS (Thr495) phosphorylation, consequently inhibiting MDA production and ONOO⁻ formation in endothelial cells. However, inhibition of AMPK reduced the anti-oxidative effects of D-4F (Figures 6, 7 and Supplementary Figure 3). Kruppel-like factor 2 (KLF2) reduces eNOS uncoupling through the Nrf2/HO-1 pathway in hypoxia/reoxygenation-induced endothelial injuries (Wu et al., 2019). D-4F upregulates HO-1 expression through the AMPK-dependent pathway (Liu et al., 2017a). Thus, AMPK might be involved in the inhibition of MDA production and ONOO⁻ formation caused by iodixanol in endothelial cells (Figures 6, 7). Collectively, D-4F might decrease iodixanol-induced apoptosis and inflammation via the AMPK/PKC-dependent inhibition on ROS production and ONOO⁻ formation in endothelial cells (Figure 9).

## Conclusion

In summary, previous study certified that D-4F improves endothelial dysfunction through scavenging excessively generated ROS. However, D-4F might exert its anti-oxidative effects through a variety of different mechanisms. This study found that iodixanol induced oxidative stress through NOX activation and ONOO⁻ formation, thus eliciting apoptosis and inflammation in endothelial cells. Nevertheless, D-4F inhibited NOX activation and eNOS dysregulation via the AMPK/PKC pathway, decreased ROS production and ONOO^−^ formation, and consequently ameliorated apoptosis and inflammation induced by iodixanol. Therefore, D-4F displayed significant anti-oxidative effects in endothelial cells through scavenging ROS and suppressing ROS generation. Hence, D-4F might serve as a potential agent to prevent CI-AKI.

## Data Availability

The raw data supporting the conclusions of this article will be made available by the authors, without undue reservation, to any qualified researcher.

## Abbreviations

AKI, acute kidney injury; AMPK, AMP-activated protein kinase; apoA-I, apolipoprotein A-I; CCK-8, cell counting kit-8; CM, contrast media; CI-AKI, contrast media–induced acute kidney injury; CVD, cardiovascular disease; DAB, 3,3-diaminobenzidine; EMPs, endothelial microparticles; eNOS, endothelial nitric oxide synthase; HDL, high-density lipoprotein; Hhcy, hyperhomocysteinemia; HO-1, heme oxygenase-1; HRP, horseradish peroxidase; HUVECs, human umbilical vein endothelial cells; ICAM-1, intercellular cell adhesion molecule-1; IL-6, interleukin-6; LDH, lactate dehydrogenase; MCP-1, monocyte chemotactic protein 1; MDA, malondialdehyde; NO, nitric oxide; NOX, NADPH oxidase; ONOO⁻, peroxynitrite; ox-LDL, oxidized low-density lipoprotein; PAI-1, plasminogen activator inhibitor-1; PKC, protein kinase C; POVPC, 1-palmitoyl-2-(5-oxovaleroyl)-sn-glycero-3-phosphocholine; rMnSOD, recombinant manganese superoxide dismutase; ROS, reactive oxygen species; SIRT1, sirtuin-1; TBARS, thiobarbituric acid reactive substances; TNF-alpha, tumor necrosis factor-alpha; t-PA, tissue-type plasminogen activator; VCAM-1, vascular cell adhesion molecule-1; vWF, von Willebrand factor.
