# Supplementary material for: D-4F Ameliorates Contrast Media–Induced Oxidative Injuries in Endothelial Cells via the AMPK/PKC Pathway
Source: Front Pharmacol. 2021 Feb 15;11:556074. doi: 10.3389/fphar.2020.556074 (PMC7917283; doi:10.3389/fphar.2020.556074)
Supplement: Supplementary file 1 [file DataSheet1.doc]

**Supplementary Methods**

**NO assay in HUVECs**

HUVECs were treated with iodixanol (30 mg I/mL) for 0, 2, 4 or 8 h. Then cells were washed twice and incubated with 4-amino-5-methylamino-2’,7’-difluorofluorescein diacetate (DAF-FM DA, D23844, Thermo Fisher Scientific, MA, USA) (2 μmol/L) for 30 min at 37℃. Fluorescence was detected through fluorescent microscopy at 495 nm excitation and 515 nm emission. The relative fluorescence intensity was analyzed using Image-Pro Plus/IOD. All experiments were repeated three times.

**Intracellular ROS assay**

HUVECs were preincubated with or without Compound C (2 μmol/L) for 1 h. Subsequently, cells were treated in the absence or presence of D-4F (20 μg/mL) for 8 h and further incubated with iodixanol (30 mg I/mL) for 6 h. Then HUVECs were washed twice with PBS and incubated with 2’,7’-dichlorofluorescindiacetate (DCFH-DA, Sigma-Aldrich, MO, USA) (2 μmol/L) for 30 min at 37 ℃. Fluorescence was detected through fluorescent microscopy at 480 nm excitation and 530 nm emission. The relative fluorescence intensity was analyzed using Image-Pro Plus/IOD. All experiments were repeated three times.

**MDA assay in the kidneys**

The tissue homogenate was prepared from frozen kidney cortex to measure MDA formation using the MDA assay kit following the manufacturer’s protocol. Protein concentrations were determined by BCA protein assay kit.

**Supplementary figure 1.**


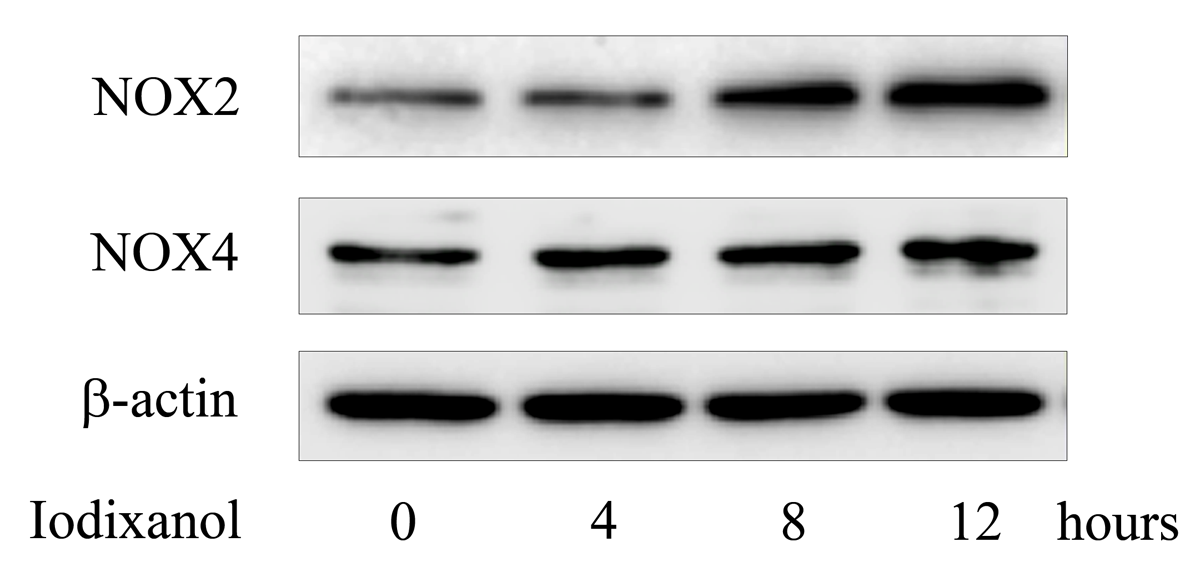


**Supplementary figure 1.** Iodixanol upregulated NOX2 and NOX4 expression in a time-dependent manner. HUVECs were treated with iodixanol (30 mg I/mL) for 0, 4, 8 or 12 h. The expression of NOX2 and NOX4 were assayed by Western blot analysis.

**Supplementary figure 2.**


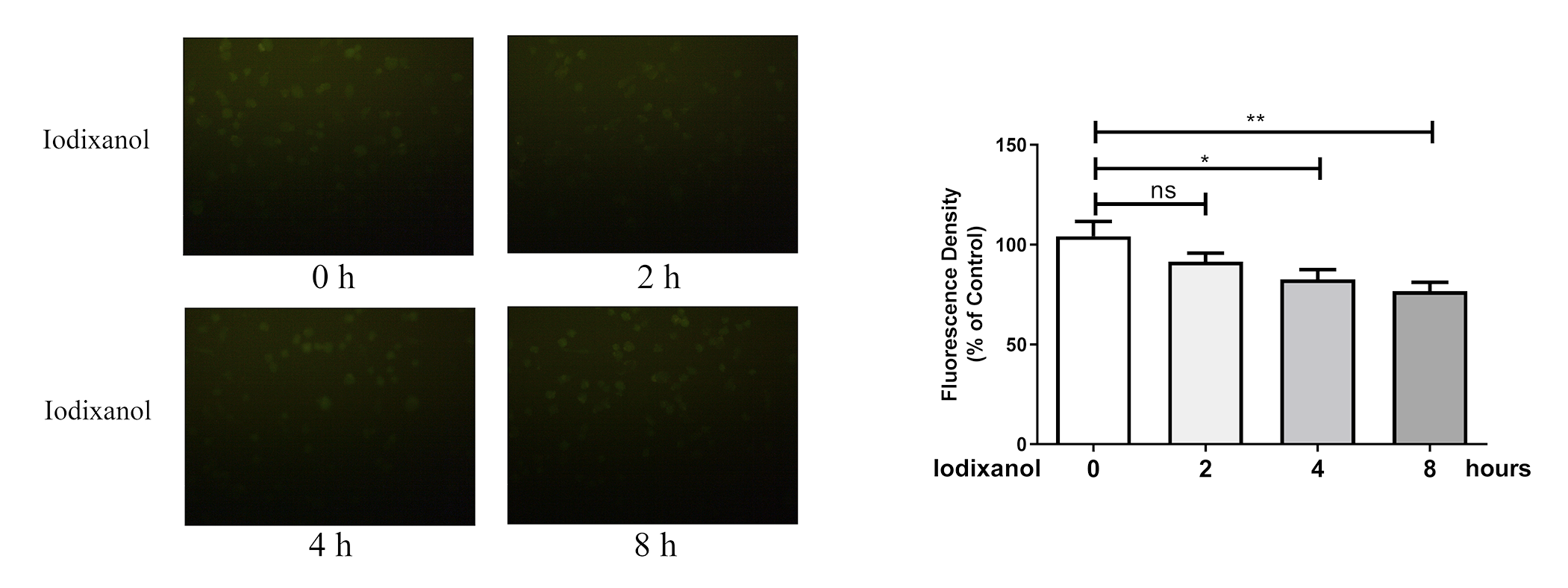


**Supplementary figure 2.** Iodixanol decreased NO production in HUVECs. HUVECs were treated with iodixanol (30 mg I/mL) for 0, 2, 4 or 8 h. NO in endothelial cells was tested through fluorescence intensity from DAF-FM DA using fluorescence microscopy (200×).

**Supplementary figure 3.**


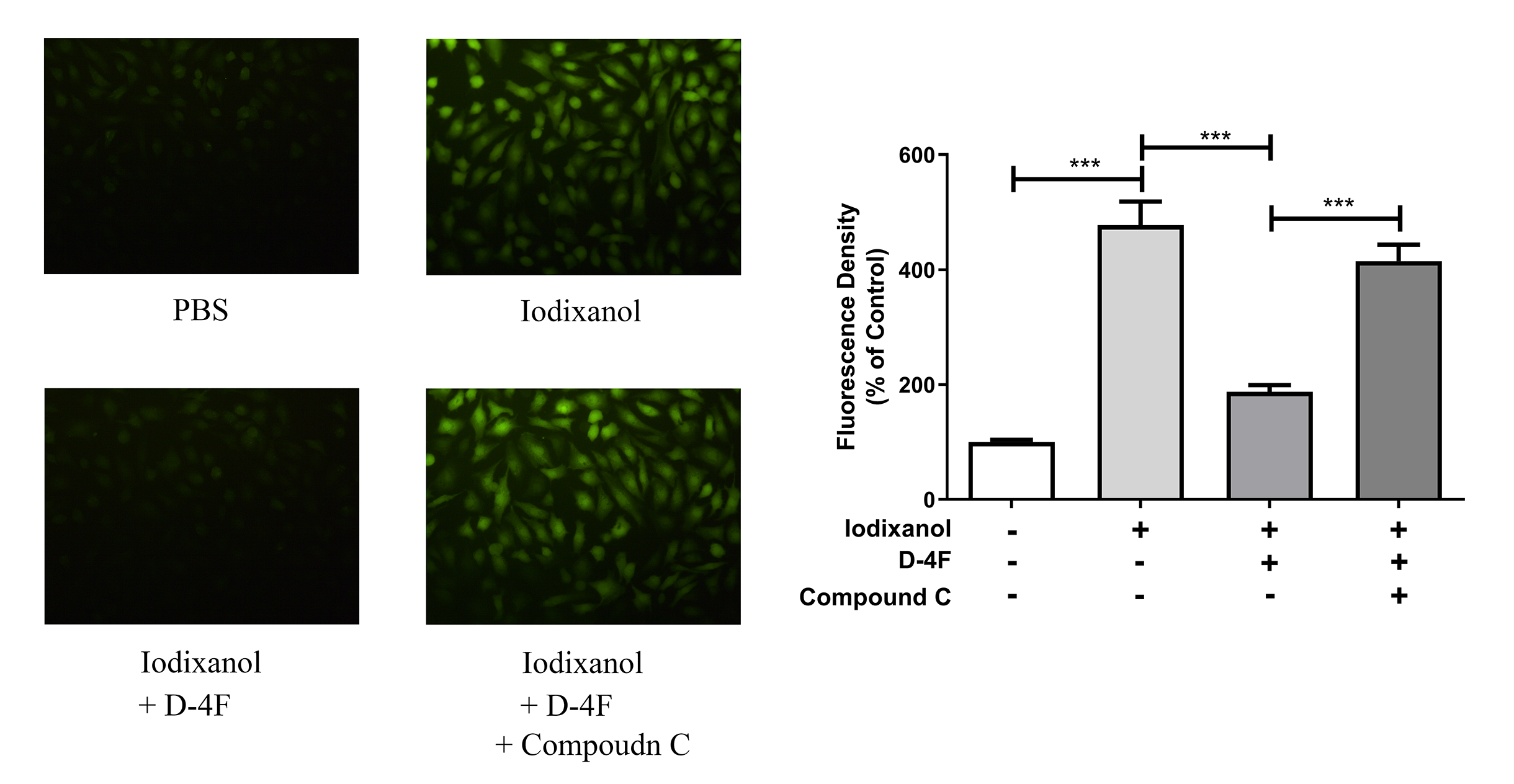


**Supplementary figure 3.** D-4F repressed iodixanol-triggered ROS release through the AMPK-dependent pathway in HUVECs. HUVECs were preincubated with or without Compound C (2 μmol/L) for 1 h and subsequently treated with D-4F (20 μg/mL) for 8 h. Then HUVECs were further incubated with iodixanol (30 mg I/mL) for 6 h. Fluorescence from DCFH-DA was detected using fluorescence microscopy (200×). The relative fluorescence intensity was analyzed using Image-Pro Plus/IOD.

**Supplementary figure 4.**

**Supplementary figure 4.** D-4F inhibited MDA formation induced by iodixanol in the kidneys *in vivo*. The kidney cortex of rats was homogenized, and the centrifuging supernatant was tested using the MDA assay kit.

**Supplementary figure 5.**


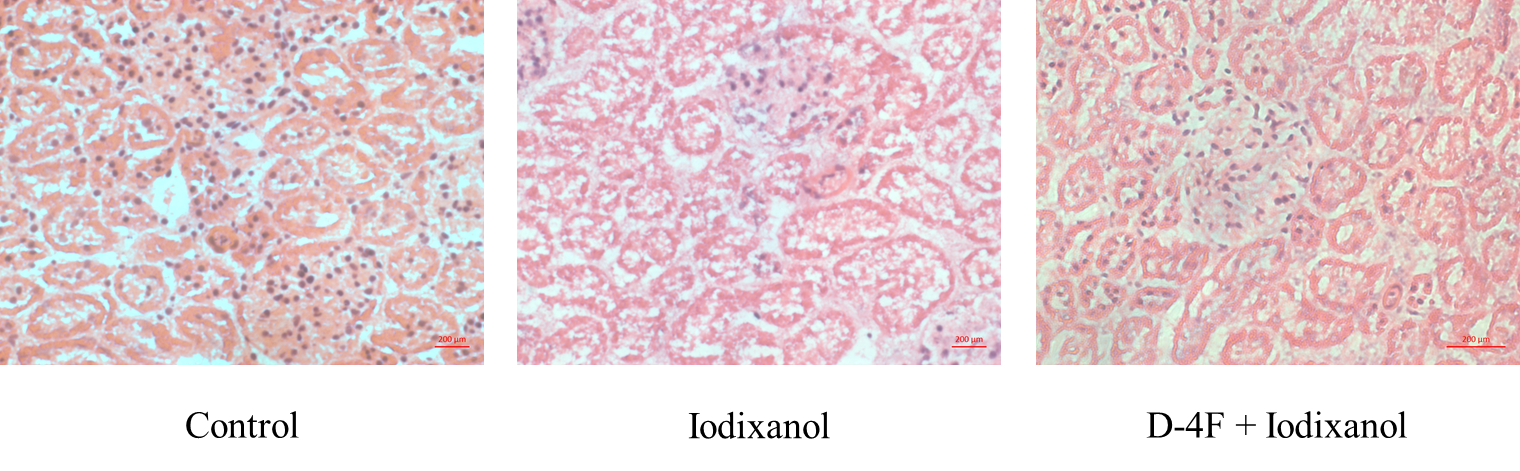


**Supplementary figure 5.** D-4F improved the morphological impairments of renal tubular cells caused by iodixanol in rats. Representative micrographs with H&E staining of the kidney sections were shown (400×).
